# A Closure Look at the Pregnancy-Associated Arterial Dissection

**DOI:** 10.3389/fcell.2021.658656

**Published:** 2021-03-12

**Authors:** Cechuan Deng, Han Wang, Xiangqi Chen, Xiaoqiang Tang

**Affiliations:** ^1^Key Laboratory of Birth Defects and Related Diseases of Women and Children of MOE, State Key Laboratory of Biotherapy, West China Second University Hospital, Sichuan University, Chengdu, China; ^2^Department of Obstetrics and Gynecology, West China Second University Hospital, Sichuan University, Chengdu, China

**Keywords:** pregnancy, arterial dissection, risk facors, diabetes, metabolism

## Introduction

Cardiovascular diseases have become the first leading cause of morbidity and mortality (Chen et al., 2020a,b; Tang et al., 2020). Cardiovascular and metabolic syndrome are common in pregnant patients (Li et al., 2017; Timpka et al., 2018). Among cardiovascular diseases, arterial dissection is a severe vascular disease with high-mortality in pregnant patients (Ramlakhan et al., 2020). Arterial dissection is a common cause of arterial disease after atherosclerosis (Adlam et al., 2016). The cause of this arterial disease is not fully understood. Analysis of the risk factors, epidemiology, and mechanisms of pregnancy-associated arterial dissections are important.

## Risk Factors of Pregnancy-associated Arterial Dissection

During the past decades, a large number of studies tried to figure out the key risk factors for pregnancy-associated arterial dissection. Hemodynamic and hormone changes during the perinatal period increase the risk factors of arterial dissection (Habashi et al., 2019; Al-Hussaini, 2020). Most previous studies have linked patients with underlying connective tissue diseases such as Marfan and Ehrlers-Danlos syndrome (Al-Hussaini, 2020). With a large population-based database of admissions, Beyer et al. provided a comprehensive analysis of the occurrence and risk factors of dissection within the peripartum period in pregnancy patients (Beyer et al., 2020). Beyer et al. statistically analyzed the occurrence of dissection during the peripartum period and explored the risk factors, time, location, and in-hospital mortality of dissection during the peripartum period. The result showed that 0.005% of pregnancy-related dissections occurred, and the most common diagnosis time point and location were during the postpartum period and coronary, respectively. The mortality of pregnant women with dissections in the hospital was higher than that of pregnant women without dissections, and the mortality of aortic dissection was the highest (8.6%). Most studies before only studied aortic dissection and spontaneous coronary artery dissection (SCAD) in pregnant women, while the relationship between arterial dissections other than aortic dissection and SCAD and pregnancy has been poorly studied. This excellent study has provided timely and comprehensive information but also left some discussion space.

## Gestational Diabetes-The Leading Risk Factor?

Based on the data of this study, gestational diabetes is the leading risk factor for pregnancy-associated arterial dissections. Interestingly, this study provided new data that gestational diabetes leads to a 65-fold increase in risk for arterial dissections during pregnancy. This finding highlights the importance of blood glucose control for pregnant patients and leaves the question that whether blood glucose intervention can reduce the risk of arterial dissections. However, previous studies revealed that diabetes mellitus is remarkably uncommon in patients with thoracic aortic dissection (LeMaire and Russell, 2011; Theivacumar et al., 2014). Besides, according to a Japan survey, aortic dissection has not occurred in women with gestational diabetes (Katsuragi et al., 2011). The risk of gestational diabetes to different types of arterial dissections may be different. A further complementary study may include this information.

## Other Confounding Factors

The authors included many complications/risk factors, and we also suggest analyzing some other risk factors. (**a**) ***Inflammatory reactions***, including systemic lupus erythematosus and multiple nodular arteritis, can increase the risk of arterial dissections in women (Tweet et al., 2018). (**b**) In addition to the Ehlers-Danlos syndrome and Marfan syndrome listed in this article, some ***other connective tissue diseases***, such as the Loeys-Dietz syndrome, are also related to arterial dissections (Ramlakhan et al., 2020). **(c)** Also, ***hereditary arterial disease*** (e.g., hereditary hypertension) is a risk factor for arterial dissections, and about 5% of female arterial dissections patients have hereditary arterial disease (Collet et al., 2020; Ramlakhan et al., 2020). Although the vast majority of the arterial dissection in the supra-aortic arteries is not related to genetic or hereditary, or acquired collagen disease, a genetic mutation in a certain component of the collagen fibers has been demonstrated, and the skin biopsy has shown some dermal dysplastic irregularity of the collagen fibers in concomitant with arterial dissection (Malfait et al., 2020). Thus, hereditary and genetic factors may contribute to arterial dissections in pregnancy patients. (**d**) Furthermore, previous studies have shown that the black race is one of the demographic characteristics and comorbid conditions of pregnancy-associated arterial dissections (Al-Hussaini, 2020; Ramlakhan et al., 2020), so ***ethnics*** can also be included as a confounding factor in the study. (**e**) Oxytocin antagonism can prevent pregnancy-associated aortic dissection in a mouse model of Marfan syndrome (Habashi et al., 2019). As thus, ***oxytocin use*** (and other drug use history) may be induced in the analysis. (**f**) Finally, other confounding factors related to arterial dissections in pregnant women include ***migraine, age at first childbirth*,** ***operation history, and infertility treatment*** (Collet et al., 2020). Including these risk factors in the further analysis would provide us more comprehensive information and improve our understanding, prevention, and treatment of pregnancy-associated arterial dissections.

## The Incidence and Mortality Trends

The authors discussed the location of arterial dissections during the peripartum period (pregnancy/delivery vs. postpartum), the total incidence and mortality of pregnancy-related arterial dissections, and the respective mortality of dissections at each site (Beyer et al., 2020). We also suggest analyzing the incidence and mortality trends of different types of dissections in pregnancy/delivery and postpartum over the course of the study period, respectively, in further study. Spontaneous cervical arterial dissection is the most common cause of ischemic stroke in North America in people younger than 45 yr (Schievink et al., 1994). The disease has a pendular behavior from mild presentations that do not require further action (like Horner's syndrome) and resolve spontaneously to devastating large hemispheric infarcts (Beletsky et al., 2003). Women with Fibromuscular dysplasia are more predisposed to present with spontaneous cervical arterial dissections and occasionally with spontaneous coronary artery dissection (Hayes et al., 2018; Gornik et al., 2019). Thus, the incidence and mortality trends, as well as risk factor information for different types of arterial dissections, are also important. The complementary analysis may provide warning information, especially for the *Center for Disease Control and Prevention*.

## Conclusion Remarks

This study elicited pregnancy and non-pregnancy risk factors that predispose to arterial dissections in all stages of pregnancy and postpartum. Further complementary analysis of detailed risk factors and for different types of pregnancy-associated arterial dissections in different periods of pregnancy would provide some more substantially basic information to this field.

## Author Contributions

CD and XT prepared and submitted the manuscript. All authors read and approved the final manuscript.

## Conflict of Interest

The authors declare that the research was conducted in the absence of any commercial or financial relationships that could be construed as a potential conflict of interest.
